# Evaluation of *Plasmodium vivax* malaria recurrence in Brazil

**DOI:** 10.1186/s12936-019-2644-y

**Published:** 2019-01-22

**Authors:** André Daher, Júlio C. A. L. Silva, Antony Stevens, Paola Marchesini, C. J. Fontes, F. O. Ter Kuile, David G. Lalloo

**Affiliations:** 1 Vice-presidency of Research and Biological Collections, Oswaldo Cruz Foundation (FIOCRUZ), Rio de Janeiro, Brazil; 2 Department of Clinical Sciences, Liverpool School of Tropical Medicine, Liverpool, UK; 3 National Institute of Infectious Disease, Oswaldo Cruz Foundation (FIOCRUZ), Rio de Janeiro, Brazil; 4 Secretariat of Health Surveillance, Ministry of Health, Brasília, Brazil; 5 Department of Transmissible Diseases Surveillance, Ministry of Health, Brasília, Brazil; 6 Faculty of Medicine, Mato Grosso Federal University, Cuiabá, Brazil

**Keywords:** Malaria, Recurrences, Plasmodium, Vivax, Falciparum, Primaquine, Chloroquine, Artemisinin-based combination therapy, ACT, Record link

## Abstract

**Background:**

Control of vivax malaria in endemic areas requires management of recurrence. The Brazilian National Malaria Surveillance System (SIVEP-Malária) records every case of malaria in Brazil, but is not designed to differentiate between primary and recurrent infections. The aim of this study was to explore whether the information provided by SIVEP-Malária could be used to identify *Plasmodium vivax* recurrences, its risk factors and evaluate the effectiveness of short course primaquine (7–9 days: total dose 3–4.2 mg/kg) in preventing relapses.

**Methods:**

In this observational retrospective cohort study, data matching of SIVEP-Malária records was undertaken using bloom filters to identify potential recurrences defined as microscopically-confirmed *P. vivax* episodes from the same individual occurring within a year. Generalized Estimation Equation (GEE) models were used to determine predictors of recurrence. Extended Cox-based conditional Prentice–Williams–Peterson models (PWP) models were used to evaluate time to recurrence.

**Results:**

Between June 1, 2014 and May 31, 2015, 26,295 episodes fulfilled the criteria of potential recurrence among 154,970 reported malaria episodes. Age ≤ 3 years, being male, literate, not-indigenous and having domestic working activities were identified as risk factors for recurrence. There was no difference in time to recurrence or recurrence frequency between patients treated with 14-day or 7–9 day primaquine regimens (HR = 1.02, 0.96–1.09) and RR = 0.97 (0.90–1.04), respectively. The use of chloroquine alone was associated with a 1.43 (1.29–1.58, p < 0.0001) increased risk of *P. vivax* recurrence compared to patients who used chloroquine combined with short-course primaquine, the Brazilian standard of care. This was RR = 2.06 (1.48–2.86, p < 0.0001), RR = 1.90 (1.60–2.25, p = 0.0001) and RR = 1.14 (1.00–1.29, p = 0.05) for recurrences occurring between 3–28, 29–60 and > 60 days, respectively. PWP models showed that the time to recurrence was longer in recipients of both primaquine and artemisinin-based combination therapy (ACT) compared to patients treated with chloroquine alone or with concomitant primaquine, HR = 2.2 (1.62–2.99, p < 0.0001), HR = 1.27 (0.97–1.66, p = 0.08), respectively.

**Conclusion:**

Short course primaquine was as effective as 14-day regimens and associated with a halving of the risk and delay in time to recurrence of *P. vivax* infections in comparison to chloroquine alone. The study demonstrates the feasibility of using record linkage on routine surveillance data to identify potential *P. vivax* recurrences, associated risk factors and impact of treatment.

**Electronic supplementary material:**

The online version of this article (10.1186/s12936-019-2644-y) contains supplementary material, which is available to authorized users.

## Background

Malaria control was first implemented in Brazil in 1905. Initial success led to the eradication campaign in 1965 [1], which lasted until the late 60s. The lowest number of annual cases, 36,900, was recorded in 1961 [2]. During the 90s, partly because of a growing population in the Amazon Region, over half a million cases per year were recorded, peaking in 1999 with 637,470 cases [3]. Since then, following the introduction of renewed malaria control efforts, including vector control and early diagnostics and treatment with artemisinin-based combination therapy (ACT) [4, 5], a drop in malaria cases has been observed with a nadir of 143,552 malaria cases in 2014. *Plasmodium vivax* infections accounted for 84% of all cases, highlighting the growing importance of this specie.

Brazil is currently focusing on the elimination of both *Plasmodium falciparum* and *P. vivax* [6, 7]. Understanding *P. vivax* recurrences is critical for malaria control in endemic areas. In Brazil, cases of malaria are recorded in the National System of Malaria Surveillance (SIVEP Malária). Although it is believed that over 99% of cases are recorded in this system, it is not designed to differentiate between primary and recurrent malaria episodes. This study aimed to explore if and how routinely collected data from the Health Surveillance System can be used to describe epidemiological patterns such as the event rate, the time interval between repeated episodes of vivax malaria, and risk factors of *P. vivax* recurrence, such as age or *P. falciparum* triggering a *P. vivax* episode.

The study also investigated the effectiveness of a 7–9 days primaquine and its comparison with a 14 days primaquine regimen, the synergic effects of concomitant use of primaquine with chloroquine and the influence of the use of ACT on the time to *P. vivax* recurrence.

## Methods

### National malaria treatment guidelines Brazil

The current first-line treatment for vivax infection is 3 days of chloroquine (600 mg on day 1, and 450 mg on days 2 and 3) with concomitant use of a short course of primaquine (7–9 days: total dose 3–4.2 mg/kg). The prescription of 14-day regimens of primaquine is indicated only if the health care provider can monitor the adherence. Pregnant women standardly receive the 3-day treatment course of chloroquine but do not receive primaquine. Only if they experience more than one episode of clinical malaria during a single pregnancy, they receive weekly chloroquine chemoprophylaxis for 12 weeks or until delivery. The current first-line treatment for uncomplicated falciparum malaria is artemether-lumefantrine [8] combined with a single dose of primaquine (maximum total dose 45 mg).

### Population and study design

This observational retrospective cohort study used records of the National System of Malaria Surveillance (SIVEP Malária) from nine States in the Brazilian Legal Amazon Region between 1st June 2014 and 31st May 2015 inclusive. The SIVEP Malaria is an online surveillance system from the Ministry of Health that records all notified cases of malaria in Brazil since 2003 [9]. This surveillance system was designed to identify outbreaks and track the effectiveness of malaria control.

It is estimated that 99.6% of malaria cases notified in Brazil were captured by the system in 2014 [5]. High rates of notification are driven by the compulsory notification of malaria in Brazil and because notification is necessary to access free malaria treatment, which is provided exclusively by the National Health System (Sistema Único de Saúde-SUS) [9] for each microscopy or rapid diagnostic tests (RDT) confirmed case of malaria. Only malaria cases in specific populations, such as illegal gold miners working in border areas, may be under represented in the National System of Malaria Surveillance.

### Recurrences and data matching

National guidelines state that any malaria positive smears conducted within 60 days of previous *P. vivax* infections and within 40 days for *P. falciparum* should be recorded as ‘follow up’ smears [8]. This information relies upon the reports provided by the patient to the health worker because the SIVEP Malaria system does not include a unique individual patient identifier, making it difficult to easily identify recurrent events.

Bloom filters matching strategies [10] were used to link records of different clinic visits made by the same patient to determine the interval in days between visits. A filter of 240 bites was defined, using a combination of patient’s name (A), patient’s mother’s name (B), date of birth (C), and municipality of residency (D) (Venn diagram presented in Additional file 1). Absent identifiers were replaced with random values to avoid unwanted similarity. The degree of similarity between two filters was assessed using the Dice Coefficient calculated on the two binary vectors [11]. The score was scaled to a maximum of 10,000. Different combinations of identifiers were used in separate reduplication runs: (a) ABCD, (b) ABD and (c) ABC. These were then individually inspected to see whether the pairs were coherent. This procedure allowed the exclusion of false matches.

### Evaluation of the population and recurrences

Baseline characteristics available from the SIVEP form (see Additional file 2) were evaluated. Age categories were matched to the primaquine dose bands of the Brazilian treatment guidelines. A map of the incidence rate of vivax recurrences (cases per 1000) at the local governmental level of the Federative Republic of Brazil (municipalities) was obtained using Tableau Desktop (version 10.3).

The effect of baseline variables on recurrent events was expressed as relative risks (RR) obtained from log-binomial regression models with random effect for patients using Generalized Estimation Equation (GEE). Time between recurrent events was stratified as 3–28, 29–60 and > 60 days based on the chloroquine half-life and per national definitions of recurrence. The same model was used to estimate the synergic effect of the concomitant use of primaquine and chloroquine on *P. vivax* recurrence.

Extended Cox-based conditional Prentice–Williams–Peterson (PWP) survival models for repeated ordered events were used for the analysis of time to vivax recurrence [12]. The time interval between sequential malaria episodes were graphically displayed using Kaplan–Meier curves and results presented as median (95% CI) time to recurrence. Two-sided p-values were used and statistical significance defined as < 0.05. Analysis was conducted using R (version 3.2.5) and IBM SPSS statistics (version 24.0).

### Ethics statement

The study protocol was reviewed and approved by the Ethics Committee at National Institute of Infectious Disease, Oswaldo Cruz Foundation (No. 1.591.434 CAAE 56245716.1.0000.5262) and complied with procedures of the Health Surveillance Secretary (SVS)—Ministry of Health of Brazil to access datasets containing personal information. The investigators ensured confidentiality of all records. The study is registered at the Brazilian Register of Clinical Trials (RBR-3n947j), a primary repository of WHO.

## Results

### Data matching results

Between June 1, 2014 and May 31, 2015 inclusive, 26,295 recurrences involving 18,185 patients were identified among 154,970 reported malaria episodes involving 128,675 patients using the Bloom Filter matching strategy. The strategy selected had ABCD, ABD and ABC Dice threshold scores of 9500. Individual record inspection identified 241 out of 17,983 matches as false matches. Some of these involved multiple potential matches between episodes from multiple patients (i.e. one visit matching to more than one patient). These 241 potential false matches were manually inspected and corrected, which resulted in 493 additional matches.

The original SIVEP Malaria dataset had 28,062 recurrent malaria episodes, however 1767 episodes were recurrences occurring within 2 days from the initial report and were excluded from the analysis giving a total of 26,295 recurrences that were used in the model (Additional file 3). The baseline characteristics are presented in Table 1.

**Table 1:** Population baseline characteristics of the study population

Variables	Categories	Patients n = 128,675 (%)	Malaria cases n = 153,203 (%)								
			Results per specie					Parasitaemia			
			Vivax	Falciparum	Mixed F + V	Malariae	Non falciparum	Less than half cross	Half to one cross	Two crosses	Three–four crosses
Age	≤ 3 years	7231 (5.62)	8584 (5.6)	690 (0.45)	34 (0.02)	0 (0)	77 (0.05)	1900 (1.24)	2791 (1.82)	4082 (2.67)	507 (0.33)
	4–8 years	9884 (7.68)	10,837 (7.07)	1060 (0.69)	54 (0.04)	1 (0)	69 (0.05)	3039 (1.98)	3799 (2.48)	4686 (3.06)	406 (0.27)
	9–11 years	6344 (4.93)	6930 (4.52)	676 (0.44)	26 (0.02)	1 (0)	41 (0.03)	2192 (1.43)	2477 (1.62)	2785 (1.82)	166 (0.11)
	12–14 years	7036 (5.47)	7637 (4.98)	680 (0.44)	42 (0.03)	0 (0)	32 (0.02)	2260 (1.48)	2866 (1.87)	3065 (2)	156 (0.1)
	15–35 years	39,704 (30.86)	41,928 (27.37)	4467 (2.92)	333 (0.22)	16 (0.01)	152 (0.1)	12,328 (8.05)	14,754 (9.64)	18,412 (12.02)	1167 (0.76)
	Older than 36 years	58,476 (45.44)	57,099 (37.27)	11,284 (7.37)	370 (0.24)	18 (0.01)	65 (0.04)	24,565 (16.04)	24,878 (16.25)	18,198 (11.88)	1069 (0.7)
Gender	Female	51,327 (39.89)	52,861 (34.5)	7658 (5)	313 (0.2)	5 (0)	197 (0.13)	19,469 (12.71)	20,956 (13.69)	19,037 (12.43)	1282 (0.84)
	Male	77,346 (60.11)	80,153 (52.32)	11,198 (7.31)	546 (0.36)	31 (0.02)	239 (0.16)	26,814 (17.51)	30,608 (19.99)	32,191 (21.02)	2189 (1.43)
Race	White	8825 (6.97)	9198 (6)	1182 (0.77)	59 (0.04)	0 (0)	21 (0.01)	2893 (1.89)	3203 (2.09)	4022 (2.63)	311 (0.2)
	African background	5024 (3.97)	4983 (3.25)	872 (0.57)	46 (0.03)	3 (0)	11 (0.01)	1548 (1.01)	1768 (1.15)	2400 (1.57)	184 (0.12)
	Asian background	1310 (1.03)	1356 (0.89)	161 (0.11)	14 (0.01)	0 (0)	2 (0)	369 (0.24)	441 (0.29)	656 (0.43)	64 (0.04)
	Mixed background	88,927 (70.25)	91,756 (59.89)	14,344 (9.36)	608 (0.4)	23 (0.02)	162 (0.11)	34,902 (22.79)	34,355 (22.44)	35,081 (22.91)	2230 (1.46)
	Native indigenous	22,492 (17.77)	23,139 (15.1)	2250 (1.47)	129 (0.08)	8 (0.01)	240 (0.16)	6119 (4)	11,017 (7.19)	7766 (5.07)	583 (0.38)
	Not informed	14,594 (11.34)	2583 (1.69)	48 (0.03)	3 (0)	2 (0)	0 (0)	453 (0.3)	781 (0.51)	1303 (0.85)	99 (0.06)
Years in School	Illiterate	77,141 (59.95)	14,523 (9.48)	2199 (1.44)	94 (0.06)	3 (0)	80 (0.05)	5721 (3.74)	5694 (3.72)	5033 (3.29)	347 (0.23)
	Incomplete/complete fundamental studies	20,725 (16.11)	78,780 (51.42)	11,958 (7.81)	534 (0.35)	28 (0.02)	236 (0.15)	27,919 (18.23)	31,415 (20.52)	30,006 (19.6)	1826 (1.19)
	Incomplete/complete high school	2227 (1.73)	22,637 (14.78)	2961 (1.93)	152 (0.1)	5 (0)	23 (0.02)	7892 (5.15)	8428 (5.5)	8889 (5.81)	531 (0.35)
	Graduation	13,988 (10.87)	1180 (0.77)	157 (0.1)	3 (0)	0 (0)	2 (0)	454 (0.3)	444 (0.29)	413 (0.27)	29 (0.02)
	Not informed	7231 (5.62)	15,895 (10.38)	1582 (1.03)	76 (0.05)	0 (0)	95 (0.06)	4298 (2.81)	5584 (3.65)	6887 (4.5)	738 (0.48)
Economic activity	Domestic	12,062 (9.37)	13,056 (8.52)	1595 (1.04)	77 (0.05)	0 (0)	27 (0.02)	4353 (2.84)	4991 (3.26)	5093 (3.33)	275 (0.18)
	Others	75,468 (58.65)	78,955 (51.54)	10,939 (7.14)	374 (0.24)	13 (0.01)	164 (0.11)	28,306 (18.49)	30,073 (19.64)	29,699 (19.4)	2092 (1.37)
	Agriculture livestock	28,192 (21.91)	28,253 (18.44)	4393 (2.87)	186 (0.12)	4 (0)	153 (0.1)	10,213 (6.67)	12,469 (8.14)	9597 (6.27)	512 (0.33)
	Tourism	2759 (2.14)	2883 (1.88)	287 (0.19)	18 (0.01)	2 (0)	8 (0.01)	649 (0.42)	1103 (0.72)	1326 (0.87)	110 (0.07)
	Mining	5999 (4.66)	5722 (3.73)	1125 (0.73)	172 (0.11)	10 (0.01)	23 (0.02)	1486 (0.97)	1334 (0.87)	3812 (2.49)	368 (0.24)
	Vegetal extractivism	1048 (0.81)	955 (0.62)	208 (0.14)	18 (0.01)	7 (0)	3 (0)	293 (0.19)	354 (0.23)	508 (0.33)	31 (0.02)
	Fishing hunting	2758 (2.14)	2758 (1.8)	282 (0.18)	10 (0.01)	0 (0)	56 (0.04)	890 (0.58)	1101 (0.72)	970 (0.63)	75 (0.05)
	Road dam construction	389 (0.3)	433 (0.28)	28 (0.02)	4 (0)	0 (0)	2 (0)	94 (0.06)	140 (0.09)	223 (0.15)	8 (0.01)

### *Plasmodium vivax* malaria recurrence

**Fig. 1:**
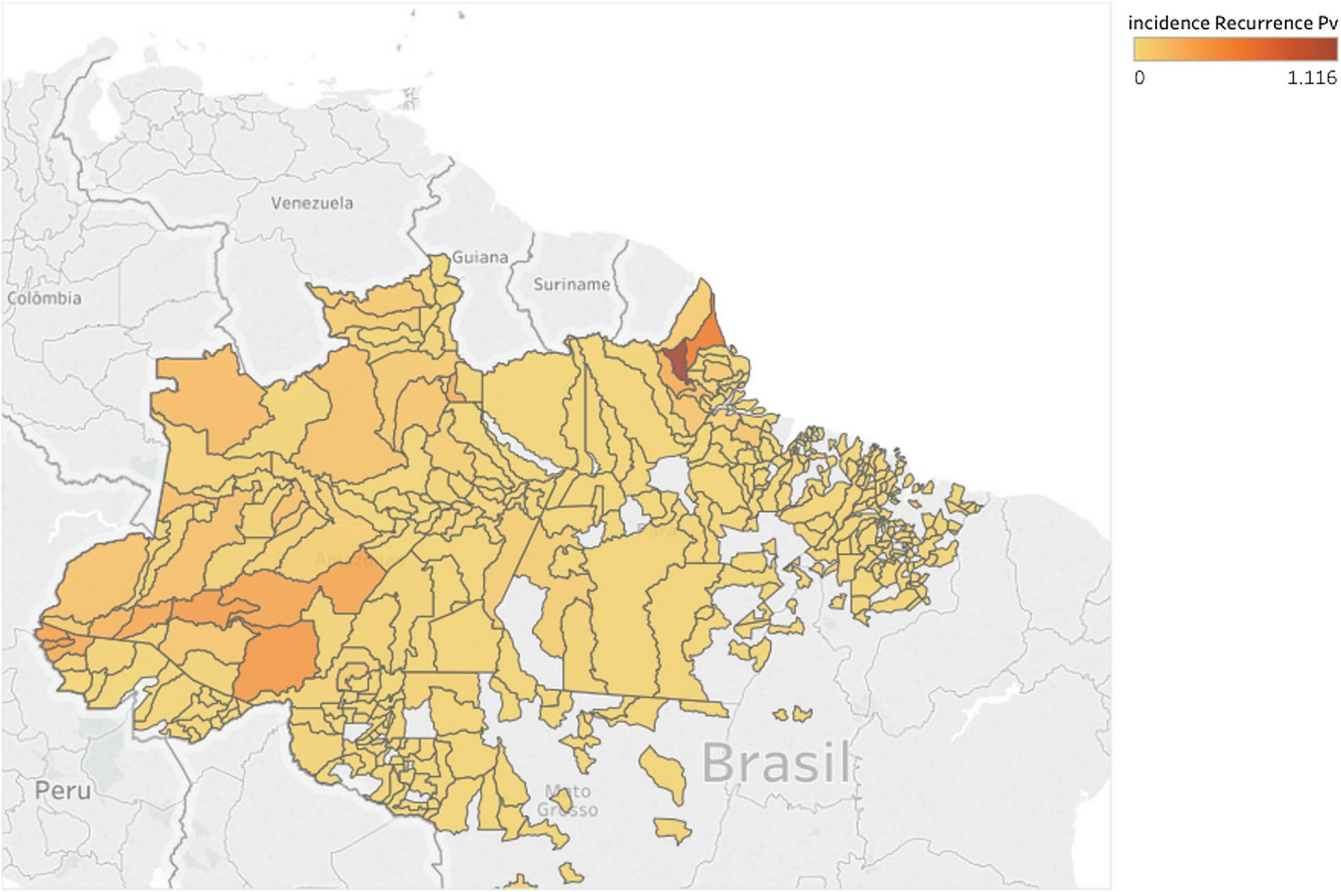
Incidence of *Plasmodium vivax* recurrence (cases per 1000)

The final combination of parameters of the strategy selected resulted in more matches than the number of recurrences reported by the health agents. The incidence rates of *P. vivax* recurrence varied considerably at the municipality level, reflecting the widespread area over which malaria transmission occurs in Brazil [13] (Fig. 1).

### Risk factors for *Plasmodium vivax* recurrences

Among those who took primaquine, the risk of recurrence decreased with increasing age. Compared to children aged **≤ **3 years, children aged 4–8 years and older adults had a 20% and 38% lower risk (4–8 years: RR = 0.80, 95% CI 0.75–0.86, p < 0.0001; adults RR = 0.62, 0.57–0.67, p < 0.0001, respectively). Being male increased the risk of *P. vivax* recurrence (RR = 1.11, 1.07–1.14, p < 0.0001). The native indigenous population were at lower risk compared to other groups. The risk of vivax recurrence was higher in all education levels compared to those who were illiterate. Individual with domestic working activities were at the highest risk of recurrence compared to other occupations.

**Fig. 2:**
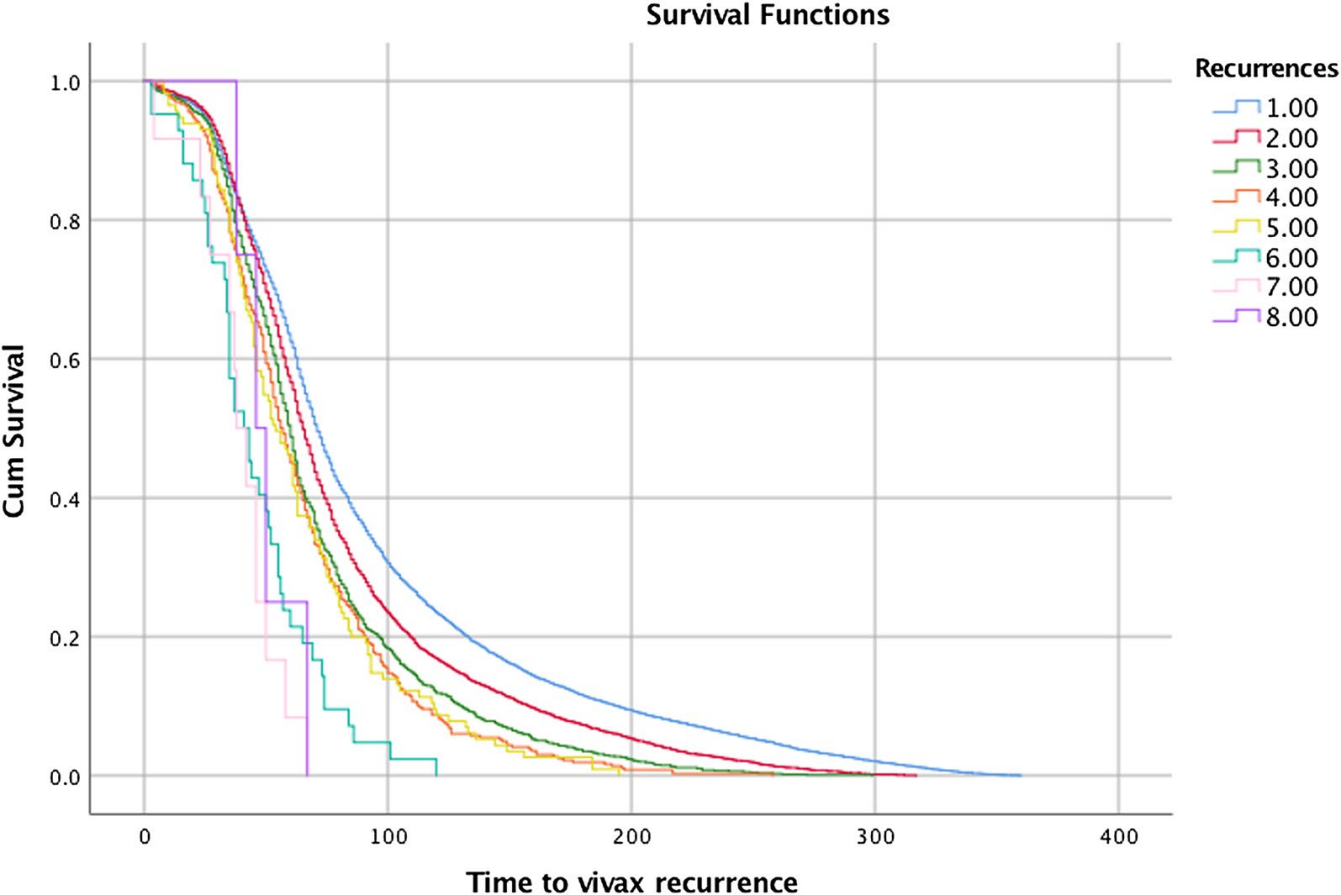
Kaplan Meier graph sequential episodes of vivax malaria

Similar risk factors were identified for time to recurrence. This reduced with each successive episode of *P. vivax* malaria (Table 2 and Fig. 2). Other factors associated with shorter time to recurrence included age ≤ 3 years, male gender, no hypnozoite treatment, and domestic occupation (Table 3). Previous *P. falciparum* infection was not associated with a shorter time to recurrence HR = 1.04, 0.93; 1.16, p = 0.50.

**Table 2:** Median time to sequential episodes of vivax malaria

Recurrences	Number of vivax recurrences	Median time (95% CI) to recurrence (days)
1st	17,460	71 (70.19–71.81)
2nd	4967	65 (63.83–66.17)
3rd	1429	60 (58.33–61.67)
4th	452	56 (52.08–59.92)
5th	150	54 (46.41–61.59)
6th	49	41 (31.47–50.53)
7th	15	38 (29.51–46.49)
8th	6	46 (34.24–57.76)
Overall		69 (68.37–69.63)

**Table 3:** Factors related to *P. vivax* recurrence

Predictor factor	Categories	Generalized Estimation Equation (GEE) model		Prentice, Williams and Peterson (PWP) survival model
		N (%) N = 151,784	RR (95% CI), p value	HR (95% CI), p value
Age categorical	Older than 36 years	68,173 (44.9)	0.62 (0.57–0.67), p < 0.0001	0.75 (0.70; 0.81), p < 0.0001
	15–35 years	46,380 (30.6)	0.71 (0.65–0.77), p < 0.0001	0.78 (0.72;0.84), p < 0.0001
	12–14 years	8337 (5.5)	0.77 (0.7–0.85), p < 0.0001	0.81 (0.74;0.88), p < 0.0001
	9–11 years	7604 (5)	0.82 (0.75–0.91), p < 0.0001	0.87 (0.80;0.96), p = 0.003
	4–8 years	11,982 (7.9)	0.8 (0.75–0.86), p < 0.0001	0.83 (0.78;0.88), p < 0.0001
	≤ 3	9308 (6.1)	REF	REF
Hypnozoite treatment	No primaquine	2667 (1.8)	1.43 (1.29–1.58), p < 0.0001	1.53 (1.39;1.69), p < 0.0001
	Primaquine 3–4.2 mg/kg	149,117 (98.2)	REF	REF
Gender	Male	90,914 (59.9)	1.11 (1.07–1.14), p < 0.0001	1.08 (1.05;1.11), p < 0.0001
	Female	60,870 (40.1)	REF	REF
Ethnic background	Not informed	1377 (0.9)	1.60 (1.4–1.82), p < 0.0001	1.19 (1.02;1.38), p = 0.03
	Native indigenous	25,705 (16.9)	0.78 (0.73–0.84), p < 0.0001	0.89 (0.84;0.95), p < 0.0001
	Mixed background	106,819 (70.4)	1.03 (0.97–1.09), p = 0.34	0.95 (0.87;1.04), p = 0.25
	Asian background	1527 (1)	0.96 (0.8–1.15), p = 0.68	0.94 (0.80;1.11), p = 0.49
	African background	5908 (3.9)	0.94 (0.86–1.04), p = 0.23	0.95 (0.87;1.04), p = 0.25
	White	10,448 (6.9)	REF	REF
Economic activity	Road dam construction	467 (0.3)	1.01 (0.8–1.27), p = 0.94	0.92 (0.74;1.13), p = 0.42
	Fishing hunting	3106 (2)	0.72 (0.63–0.82), p < 0.0001	0.73 (0.65;0.82), p < 0.0001
	Vegetal extraction	1191 (0.8)	0.59 (0.48–0.73), p < 0.0001	0.70 (0.58;0.85), p < 0.0001
	Mining	7046 (4.6)	0.81 (0.74–0.89), p < 0.0001	0.86 (0.79;0.93), p < 0.0001
	Tourism	3182 (2.1)	0.79 (0.71–0.89), p < 0.0001	0.76 (0.68;0.84), p < 0.0001
	Agriculture livestock	32,919 (21.7)	0.77 (0.73–0.82), p < 0.0001	0.79 (0.75;0.83), p < 0.0001
	Others	89,132 (58.7)	0.85 (0.81–0.9), p < 0.0001	0.91 (0.87;0.95), p < 0.0001
	Domestic	14,741 (9.7)	REF	REF
Years in school	Complete graduation	1341 (0.9)	1.14 (0.97–1.35), p = 0.12	1.04 (0.89;1.22), p = 0.63
	Complete high school	13,647 (9)	1.14 (1.06–1.23), p < 0.0001	1.07 (1.00;1.15), p = 0.04
	Incomplete high school	12,070 (8)	1.15 (1.07–1.24), p < 0.0001	1.09 (1.02;1.17), p = 0.02
	Complete fundamental studies	9745 (6.4)	1.11 (1.03–1.2), p = 0.01	1.05 (0.97;1.13), p = 0.24
	Not informed	17,596 (11.6)	1.33 (1.22–1.44), p < 0.0001	1.27 (1.17;1.37), p < 0.0001
	Incomplete fundamental studies	81,190 (53.5)	1.12 (1.06–1.18), p < 0.0001	1.06 (1.01;1.12), p = 0.02
	Illiterate	16,195 (10.7)	REF	REF

*REF* reference group

### Effectiveness of 7 days versus 14 days primaquine on *P. vivax* recurrence incidence

Overall, 6226 (4.9%) patients were prescribed a 14-day course of primaquine and 120,608 (95.1%) a 7-day course (total dose 3–4.2 mg/kg). All received 3-days of chloroquine for *P. vivax* malaria. The risk of *P. vivax* recurrence was similar between the two regimens: 14-day: 887/6226 (14.3%) vs 7–9 day: 17,610/12,0608 (14.6%), RR = 0.97 (0.90–1.04), p = 0.96. Similar conclusions could be drawn from the PWP survival model which also showed no difference in the time to recurrence (HR = 1.02, 0.96–1.09, p = 0.52).

### Effectiveness of the concomitant use of primaquine on *P. vivax* recurrence incidence

As stated previously, primaquine treatment is not recommended in Brazil for pregnant women and chloroquine chemoprophylaxis for pregnant women is only given after the second malaria episode. This enabled the use of the pregnant population with a single vivax recurrence as a comparator to assess the effect of hypnozoite treatment.

Pregnant patients who did not take short-course primaquine had a 1.43 (95% CI [1.29–1.58]) risk of *P. vivax* recurrence compared to non-pregnant patients who took primaquine. This was RR = 2.06 (1.48–2.86, p < 0.0001), RR = 1.90 (1.60–2.25, p = 0.0001) and RR = 1.14 (1.00–1.29, p = 0.05) for recurrences occurring between 3–28, 28–60 and > 60 days. The time to a *P. vivax* recurrence was also shorter in pregnant women (HR = 1.53, 1.39–1.69, p < 0.0001).

Overall, 399 patients received ACT with a primaquine total dose of 3–4.2 mg/kg starting after the third day of ACT to treat mixed *P. vivax* and *P. falciparum* infections. There was a reduced risk of *P. vivax* recurrence in those taking ACT and sequential primaquine compared to pregnant patients treated with chloroquine only (RR = 0.70, 0.52–0.93, p = 0.015). *Plasmodium vivax* recurrence also took longer in patients taking ACT and sequential primaquine than chloroquine alone treated patients HR = 2.2 (1.62–2.99, p < 0.0001), and those treated with chloroquine and with concomitant primaquine, HR = 1.27 (0.97–1.66, p = 0.08), but this difference was of borderline statistical significance.

## Discussion

This study demonstrated the potential for the use of routinely collected public health surveillance records to evaluate the epidemiology and risk factors of recurrent *P. vivax* malaria in Brazil. The computational tools available allowed the processing and successful automated matching of over 150,000 case records to identify potential recurrent infections. This provided access to unique data that allowed us to identify associated risk factors and impact of treatment using routine surveillance data. The analysis showed that short 7–9 day courses of primaquine with concomitant use of chloroquine was more effective than chloroquine alone, and equally as effective as the 14-day regimen in preventing *P. vivax* recurrences. ACT-primaquine combinations were also more effective than chloroquine alone or chloroquine–primaquine combinations in delaying recurrence, although the latter was not statistically significant.

An important part of this analysis included matching of case records in the absence of a unique patient identifier to link different episodes that occurred in the same individual during a 1-year period. Although there are limitations in the quality of the routine data in many surveillance systems in resource poor settings, using computational approaches to explore the large datasets may help generate evidence to support public health policies [14]. Bloom filters are designed to screen vast amounts of data with applications in many fields of computational science [15] and are aimed at detecting duplicate entries. The quality of data entry is a major factor in defining the best matching strategy and, therefore, there is no standard strategy suitable for all datasets. This meant that, depending on the completeness of the dataset using high Dice’s coefficients in the matching strategy led to a low number of matches (low sensitivity). However, the visual inspection of the lower thresholds had too many false matches (low specificity). The current strategy identified a greater frequency of recurrences than the one reported by the health agents based on patient’s recall.

Brazil recommends a short 7–9 days courses (total dose 3–4.2 mg/kg). Fourteen days of primaquine is only recommended in Brazil either when patients are considered likely to be compliant or can undergo supervised treatment. The similar effectiveness between these two regimens is encouraging, reinforcing the idea that cure rate is a function of the total primaquine dose rather than treatment length [16, 17].

The importance of addressing the effectiveness of primaquine in real life for radical cure of *P. vivax* infections was highlighted recently [18]. This equal effectiveness of the short-course regimen has important practical implications because a patient friendly primaquine regimen is desirable and regimens of shorter duration are more likely to be adhered to [19]. The evidences on which this short-course primaquine regimen in Brazil is based on are a limited number of clinical trials [20, 21], programmatic experience, and historical evidence suggesting that the efficacy of primaquine is more a function of the cumulative dose than the duration of treatment [17]. However, the evidence has been deemed insufficient to support international guidelines. An ongoing systematic review [22] and the results of this current analysis may increase this body of evidence.

Children under 3 years old had an increased risk of recurrence compared to all other age categories, with a trend towards increasingly lower risk with increasing age. There are several potential explanations for this in addition to differences in acquired immunity: The doses of chloroquine and primaquine may be inadequate at this young age [23] or the absence of child-friendly formulations may result in poor adherence and reduced effectiveness. Herein it was demonstrated a reduced risk of recurrence in the native indigenous population. Similarly, it was also showed that the risk was lowest in the illiterate population. Both may reflect higher exposure rates early in life and a more rapid acquisition of protective immunity in these groups [24].

Previous falciparum infection was not found to caused more rapid recurrence of *P. vivax*, in contrast to suggestions from others in the literature [24]. It is not clear if this is a function of a long duration of post-treatment prophylactic effect of ACT to suppress *P. falciparum* infections.

It was found that the time to recurrence appears to reduce with each subsequent episode of vivax recurrence. Although these observations are in contrast with the current understanding of hypnozoite activation which implies a gradual lengthening, rather than shortening with each successive relapse, these results are consistent with previous observations in Brazil [25]. The underlying assumption related to the gradual lengthening hypothesis is that with multiclonal infections the earliest active and more rapidly multiplying parasite became patent first [24]. These observations relied on artificial infection and animal models. Although this study cannot differentiate with relapse, reinfections and recrudescence, it does not provide strong support for a gradual lengthening of relapse intervals. Further research to address the issue is needed.

The observation that the addition of primaquine was already effective in preventing recurrence in the first 28 days is interesting. It implies that the addition of primaquine to chloroquine either reduced recrudescence or early relapses and may support the synergic effect of primaquine and chloroquine in the blood stage [26–29]. This trend is also seen in the following period until the 60th day. A small (RR = 1.14) effect was also seen beyond 60 days, but it is not clear if this reflects a long-lasting anti-relapse effect of primaquine [4] or a higher exposure risk in the non-primaquine recipients resulting in more new infections and thus a higher rate of recurrence.

The study design is an important limitation. This is a 1-year retrospective open cohort, meaning that patients infected before the study start yet recurring during the study were considered as initial events. Second, the only way that the effectiveness of short course of primaquine could be evaluated was to use the pregnant population as the reference group because this group does not receive primaquine during pregnancy. However, this is a biased population and the results must be interpreted with this in mind. Potential confounders include changes in mosquito biting frequency during pregnancy, altered personal protection and healthcare-seeking behaviour, altered immunological responses, and changes in distribution of drugs, although chloroquine pharmacokinetics parameters in pregnant women does not appear to be very different from the rest of the population [30]. Despite these limitations, our results do suggest that the current national treatment guidelines in Brazil are effective in reducing relapse.

Additionally, every data matching strategy has its limitations and they can result in different number of recurrences depending on the reliability of the data entry. A unique identifier for the patients would allow a better evaluation of the sensitivity and specificity of the strategy and increase the use of the database as routine. Besides the data quality concerns, microscopy does not allow differentiation between relapse, recrudescence and reinfection of the *P. vivax* episodes and the aggregate recurrence was used in the analysis. Routine genotyping to differentiate these is currently not feasible at scale under programmatic conditions. Efforts to differentiate relapse and reinfection in this study by restringing the analysis to infants born during the study and, therefore, free of hypnozoites, was not successful as the sample of newborns was too small. Multi-year cohorts may address this issue.

## Conclusions

Overall, this study confirmed that under real-life conditions, short-course primaquine was equally as effective as the 14-day regimen, which is the standard in many other countries. ACT-primaquine combinations were also more effective than chloroquine alone or chloroquine-primaquine combinations in delaying recurrence. The concomitant use of primaquine and chloroquine either reduced recrudescence or very early relapse and may support their synergic effect in the blood stage. Finally, the study demonstrates the feasibility of using record linkage to identify potential *P. vivax* recurrences, associated risk factors and impact of treatment using routine surveillance data. These routine data could also potentially provide a baseline for evaluation of further malaria control interventions that address the malaria control in Brazil.

## Additional files

**Additional file 1.** Venn Diagram of record linkage.

**Additional file 2.** SIVEP form.

**Additional file 3.** Data flowchart.

## Abbreviations

ACT: artemisinin-based combination therapy
FIOCRUZ: Oswaldo Cruz Foundation
GEE: Generalized Estimation Equation models
HR: Hazard Ratio
PWP: Prentice–Williams–Peterson models
RDT: rapid diagnostic tests
RR: relative risk
SIVEP-Malária: Brazilian National Malaria Surveillance System
