# Supplementary material for: Evaluation of Plasmodium vivax malaria recurrence in Brazil
Source: Malar J. 2019 Jan 22;18:18. doi: 10.1186/s12936-019-2644-y (PMC6343355; doi:10.1186/s12936-019-2644-y)
Supplement: Supplementary file 1 — Additional file 1. Venn Diagram of record linkage. [file 12936_2019_2644_MOESM1_ESM.docx]

Venn Diagram of record linkage

A

(Name)

B (Mother’s name)

C (DOB)

D

(Municipality)

AC

BC

AD

AB

ACD

BD

ABC

BCD

ABD

ABCD

CD

(I)

(II)

(V)

(III)

(IV)
