# Supplementary material for: Evaluation of Plasmodium vivax malaria recurrence in Brazil
Source: Malar J. 2019 Jan 22;18:18. doi: 10.1186/s12936-019-2644-y (PMC6343355; doi:10.1186/s12936-019-2644-y)
Supplement: Supplementary file 2 — Additional file 2. SIVEP form. [file 12936_2019_2644_MOESM2_ESM.pdf]

República Federativa do Brasil
Ministério da Saúde

SIVEP
SISTEMA DE INFORMAÇÕES DE VIGILÂNCIA EPIDEMIOLÓGICA
NOTIFICAÇÃO DE CASO MALÁRIA

1 Nº da Notificação:

DADOS DA NOTIFICAÇÃO

2 Data da Notificação:

3 Tipo de Detecção: **1**-Passiva **2**-Ativa

4 Sintomas: **1**-Com sintomas **2**-Sem sintomas

5 UF Notificação:

6 Município da Notificação:

7 Cód.Mun. Notificação:

8 Unidade Notificante:

9 Código da Unidade:

10 Nome do Agente Notificante:

11 Código do Agente:

DADOS DO PACIENTE

12 Nome do Paciente:

13 Nº Cartão Nacional de Saúde:

14 Data de Nascimento:

15 Idade: Dia Mes Ano

16 Sexo: **M**- Masculino **F**- Feminino

17 Paciente é gestante? **1**-1º Trimestre **2**-2º Trimestre **3**-3º Trimestre **4**-Idade gestacional ignorada **5**-Não **6**-Não se aplica

18 Escolaridade: **0**-Analfabeto **1**-1ª a 4ª série incompleta do EF **2**-4ª série completa do EF **3**-5ª a 8ª série incompleta do EF **4**-Ensino fundamental completo **5**-Ensino médio incompleto **6**-Ensino médio completo **7**-Educação superior incompleto **8**-Educação superior completa **10**-Não se aplica

19 Raça/Cor: **1**-Branca **2**-Preta **3**-Amarela **4**-Parda **5**-Indígena

20 Nome da mãe:

21 Principal Atividade nos Últimos 15 Dias: **1**-Agricultura **2**-Pecuária **3**-Doméstica **4**-Turismo **5**-Garimpagem **6**-Exploração vegetal **7**-Caça/pesca **8**-Construção de estradas/barragens **9**-Mineração **10**-Viajante **11**-Outros

22 Endereço do Paciente:

23 Outro País de Residência:

24 UF Residência:

25 Município de Residência:

26 Cód. Mun. Resid:

27 Localidade de Residência:

28 Cód.Localid. Resid:

29 Data dos Primeiros Sintomas:

30 Recebeu tratamento para malária vivax nos últimos 60 dias? **1**-Sim **2**-Não

31 Recebeu tratamento para malária falciparum nos últimos 40 dias? **1**-Sim **2**-Não

LOCAL PROVÁVEL DA INFECÇÃO

32 Outro País Provável de Infecção:

33 UF Provável de Infecção:

34 Município Provável de Infecção:

35 Cód. Mun. Provável Infecção:

36 Localidade Provável de Infecção:

37 Cód. Localid. Prov. Infecção:

DADOS DO EXAME

38 Data do Exame:

39 Tipo de exame: **1**-Gota espessa/Esfregaço **2**-Teste rápido

40 Resultado do Exame: **1**- Negativo; **2**- F; **3**- F+FG; **4**- V; **5**- F+V; **6**- V+FG; **7**- FG; **8**- M; **9**- F+M; **10**- Ov; **11**-Não F

41 Parasitos por mm^3:

42 Parasitemia em "cruzes".: **1**- < +/2 (menor que meia cruz); **2**- +/2 (meia cuz); **3**- + (uma cruz); **4**- ++ (duas cruzes); **5**- +++ (três cruzes); **6**- ++++ (quatro cruzes)

43 Outros Hemoparasitos Pesquisados: **1**-Negativo **2**-Trypanosoma sp. **3**-Microfilária **4**-Trypanosoma sp.+Microfilária **9**-Não pesquisados

44 Nome do Examinador:

45 Cód Examinador:

TRATAMENTO

46 Esquema de tratamento utilizado, de acordo com Manual de Terapêutica da Malária

1- Infecções pelo P. vivax ou P. ovale com cloroquina em 3 dias e primaquina em 7 dias (esquema curto);
2- Infecções pelo P. vivax, ou P. ovale com cloroquina em 3 dias e primaquina em 14 dias (esquema longo);
3- Infecções pelo P. malariae para todas as idades e por P. vivax ou P. ovale em gestantes e crianças com menos de 6 meses, com cloroquina em 3 dias;
4- Prevenção das recaídas frequentes por P. vivax ou P. ovale com cloroquina semanal em 12 semanas;
5- Infecções por P. falciparum com a combinação fixa de artemeter+lumefantrina em 3 dias;
6- Infecções por P. falciparum com a combinação fixa de artesunato+mefloquina em 3 dias;
7- Infecções por P. falciparum com quinina em 3 dias, doxiciclina em 5 dias e primaquina no 6º dia;
8- Infecções mistas por P. falciparum e P. vivax ou P. ovale com Artemeter + Lumefantrina ou Artesunato + Mefloquina em 3 dias e Primaquina em 7 dias;
9- Infecções não complicadas por P. falciparum no 1º trimestre da gestação e crianças com menos de 6 meses, com quinina em 3 dias e clindamicina em 5 dias;
10- Malária grave e complicada pelo P. falciparum em todas as faixas etárias;
11- Infecções por P. falciparum com a combinação fixa de artemeter+lumefantrina em 3 dias e primaquina em dose única;
12- Infecções por P. falciparum com a combinação fixa de artesunato+mefloquina em 3 dias e primaquina em dose única;
99- Outro esquema utilizado (por médico) - descrever:

47 Data Início do Tratamento:

SMS-UF MUNICÍPIO

12 Nome do Paciente:

15 Idade:

1 Nº da Notificação

38 Data do Exame

40 Resultado do Exame

44 Nome do Examinador:

Comprovante de resultado do exame para ser entregue ao paciente

MS/SVS 28/02/2014
