# Supplementary material for: Evaluation of Plasmodium vivax malaria recurrence in Brazil
Source: Malar J. 2019 Jan 22;18:18. doi: 10.1186/s12936-019-2644-y (PMC6343355; doi:10.1186/s12936-019-2644-y)
Supplement: Supplementary file 3 — Additional file 3. Data flowchart. [file 12936_2019_2644_MOESM3_ESM.docx]

Data flowchart

Visual inspection

SIVEP malaria dataset

154,970

(records)

128,675

patients

Data matching

17,983

Patients with recurrences

26,295 recurrences

(records)

1,767 recurrences until D2

153,203

(records)

291

False matches

493

New matches
